# Differential effects of extracellular vesicles from aging and young mesenchymal stem cells in acute lung injury

**DOI:** 10.18632/aging.102314

**Published:** 2019-09-29

**Authors:** Ruoqiong Huang, Chaojin Qin, Jiangmei Wang, Yaoqin Hu, Guoping Zheng, Guanguan Qiu, Menghua Ge, Huikang Tao, Qiang Shu, Jianguo Xu

**Affiliations:** 1 Children’s Hospital, Zhejiang University School of Medicine, Hangzhou, Zhejiang 310052, China; 2 Shaoxing Second Hospital, Shaoxing, Zhejiang 312000, China; 3 First Affiliated Hospital, Zhejiang University School of Medicine, Hangzhou, Zhejiang 310003, China

**Keywords:** aging, mesenchymal stem cells, ARDS, acute lung injury, extracellular vesicles

## Abstract

Old age is a known risk factor for mortality in acute respiratory distress syndrome (ARDS)/acute lung injury. Mesenchymal stem cells (MSCs) possess potent immunomodulatory properties, while aging MSCs have reduced capacity. Recent studies have demonstrated that MSC-derived extracellular vesicles (MSC-EVs) are able to mimic MSCs in alleviating acute lung injury. The goals of this study were to determine whether EVs from young and aging MSCs had differential effects on lipopolysaccharide (LPS)-induced lung injury in young mice and unravel the underlying mechanisms. Our results showed that both aging and young MSC-EVs had similar physical and phenotypical properties. As their parental cells, young MSC-EVs alleviated LPS-induced acute lung injury, while aging MSC-EVs did not exhibit the protective effects. In contrast to young MSC-EVs, aging MSC-EVs failed to alter macrophage phenotypes and reduce macrophage recruitment. In addition, the internalization of aging MSC-EVs by macrophages was significantly lower compared with that of young MSC-EVs. Furthermore, aging and young MSC-EVs differed in levels of several miRNAs relating macrophage polarization. In conclusion, aging and young MSC-EVs have differential effects in alleviating acute lung injury and macrophage polarization, which may be associated with internalization of EVs and their miRNA content.

## INTRODUCTION

Acute respiratory distress syndrome (ARDS)/acute lung injury, which is characterized by acute hypoxemia and bilateral opacities on chest radiograph, is one of the common complications of critical illnesses and a life-threatening lung disease [1]. Hospital mortality of ARDS is still at a high rate of 30%~40%, even though treatments for ARDS have improved recently, including extracorporeal carbon dioxide removal, high-frequency oscillatory ventilation, lung recruitment maneuver, and prone positioning [2]. ARDS is the result of direct or indirect lung injury, precipitated by factors such as pneumonia, sepsis, aspiration of gastric contents, and major trauma. During lung injury, resident macrophages are activated and skewed toward an M1 activation state with the secretion of proinflammatory cytokines. Subsequently, neutrophils are recruited to promote the inflammatory responses. The severe inflammatory responses lead to lung endothelial injury, alveolar epithelial injury, and the accumulation of protein-rich fluid in the alveolar space [3]. At present, there is no truly effective pharmacologic therapy for ARDS [4]. There is an urgent need for novel therapeutic approaches to improve the outcome of ARDS. Treatment with mesenchymal stem cells (MSCs) might be such an ideal approach. MSCs exert their immunomodulatory effects via inhibiting proliferation and activity of natural killer cells, suppressing proliferation and activation of T and B lymphocytes, and blocking maturation of dendritic cells. In addition, MSCs are able to induce the expansion of regulatory T cells [5]. Many studies, including the previous work from our lab [6], have elucidated that MSCs are capable of alleviating acute lung injury in animal models [7]. In addition, the protective effects occurred without significant engraftment of MSCs to the lung tissue. Furthermore, our group [8] and others [9] have demonstrated that MSCs are safe in ARDS patients. Current body of literature supports a paracrine mechanism of MSCs, which are mediated by both soluble paracrine factors and MSC-derived extracellular vesicles (MSC-EVs) [10]. Morrison et al. demonstrated that MSCs favored an anti-inflammatory M2 macrophage phenotype via MSC-EV-mediated mitochondrial transfer. Adoptive transfer of macrophages pretreated with MSC-EVs ameliorated LPS-induced acute lung injury in a mouse model [11].

EVs are a heterogeneous population of membrane vesicles released by almost all types of cells. These vesicles contain molecular components such as proteins, DNA, mRNA, and microRNAs (miRNAs) [12]. They were initially thought to be unwanted cellular debris and are now recognized as a new mechanism of intercellular communication and biomarkers for diseases [13]. Based on their origins and sizes, EVs are further classified into three subclasses: exosomes (30–150 nm), microvesicles (100–1000 nm), and apoptotic bodies (1–4 μm) [14]. MSC-EVs are characterized by the expression of the surface markers for MSCs such as CD29, CD73, CD90, CD44, and CD105, along with the markers for EVs such as CD107, CD63, CD9 and CD81 [15]. Several studies have reported that MSC-EVs are able to mimic MSCs in alleviating acute lung injury. In a mouse model of lipopolysaccharide (LPS)-induced acute lung injury, MSC-EVs reduced lung edema, protein permeability, and neutrophil infiltration. The effects of MSC-EVs were partially blocked by keratinocyte growth factor (KGF) siRNA, indicating the involvement of KGF mRNA [16]. In a mouse model of E. coli-induced pneumonia, MSC-EVs enhanced animal survival, alleviated lung inflammation, and decreased bacterial load. The mechanism involves the uptake of EVs through the CD44 receptor by monocytes and alveolar epithelial cells [17].

Aging involves alteration in physiological and biological function. The immune system in older age is in a state of immunosenescence. Both innate immune response and adaptive immune response are underperformed in the elderly [18]. Another hallmark of aging is exhaustion and functional decline of stem cells, including MSCs [19]. These alterations in aging contribute to the increased vulnerability to critical illnesses in old age, such as ARDS. Tashiro et al. reported that young MSCs but not aging MSCs inhibited bleomycin-induced pulmonary fibrosis in aged mice. Compared with aging MSCs, young MSCs had lower mRNA expression of metalloproteinase-2 and insulin-like growth factor receptor [20]. Bustos et al. documented that aging MSCs failed to protect endotoxemia-induced lung injury compared with young counterparts. They demonstrated that the expression of cytokine and chemokine receptors was age-dependent and essential for the migration and activation of MSCs [21].

In the present study, we aimed to compare the protective effects between young and aging MSC-EVs in acute lung injury and elucidate the potential mechanisms. MSC-EVs were isolated from health donors of 25 and 72 years old, respectively, and administered to young mice with LPS-induced lung injury.

## RESULTS

### Aging MSCs have impaired therapeutic effects in acute lung injury

**Figure 1:**
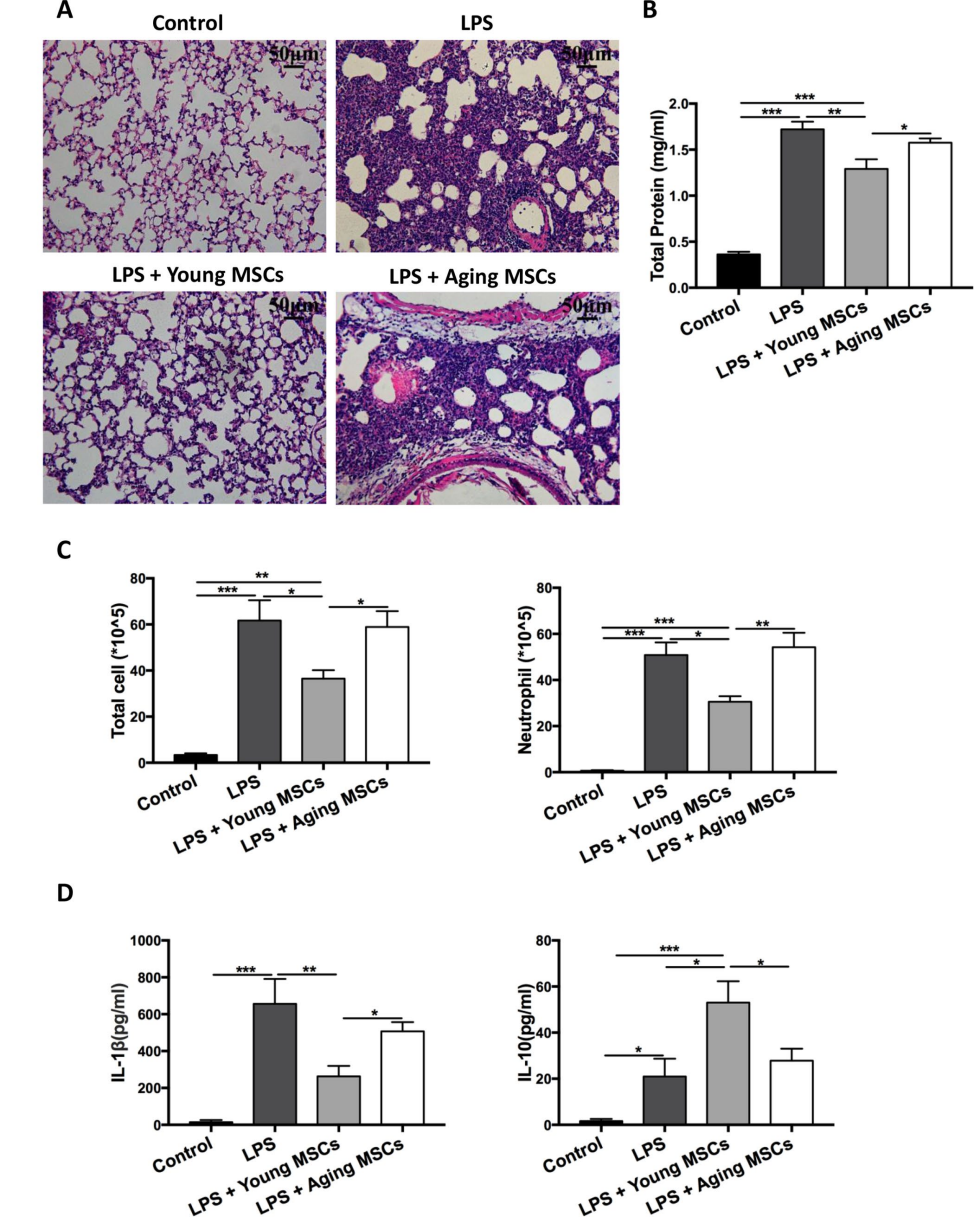
**Aging MSCs showed no beneficial effects in LPS-induced lung injury compared with young MSCs.** Mice were divided into 4 group: control, LPS, LPS + young MSCs (1 × 10^6^ cells), LPS + aging MSCs (1 × 10^6^ cells). MSCs were administered intravenously 30 min after intratracheal LPS treatment. (**A**) Lung tissue was harvested at 48 h after LPS treatment and stained with H&E and visualized at x200 magnification (scale bar: 50 μm). (**B** and **C**) Protein level, total cell count, and neutrophil count in the BAL were examined at 48 h after LPS treatment to evaluate inflammatory response. Data are presented as mean ± SEM, n = 7–10. *p < 0.05, **p < 0.01, *** *p* < 0.001. (**D**) Cytokine levels (IL-1β and IL-10) in the BAL at 48 h after LPS treatment were assayed via ELISA. Data are presented as mean ± SEM, n = 4–7. *p < 0.05, **p < 0.01, *** *p* < 0.001.

In a mouse model of acute lung injury induced by endotoxemia, Bustos et al. showed that aging MSCs lacked the anti-inflammatory effects [21]. To mimic the human ARDS condition, acute lung injury was induced via intratracheal administration of LPS to young C57BL/6 mice (6~8 weeks old) and compared after administering young or aging MSCs. Lung histology at 48 h after LPS insult showed that adoptive transfer of young MSCs reduced the influx of inflammatory cells and thickening of alveolar septum compared with the LPS treatment, while aging MSCs lost the beneficial effects (Figure 1A). Compared with LPS-treated mice, young MSCs significantly decreased lung permeability, as reflected by reduced protein level in the bronchoalveolar lavage (BAL) at 48 h after LPS treatment, by 25.0% (Figure 1B). Young MSCs also reduced the number of total cells by 40.9% and neutrophils by 39.9% compared with LPS treatment (Figure 1C). In addition, young MSCs lowered proinflammatory cytokine IL-1β and elevated anti-inflammatory IL-10 (Figure 1D). In contrast, the anti-inflammatory effects of aging MSCs were significantly decreased compared with young counterparts (Figure 1B–1D).

### Aging and young MSCs-EVs have similar physical and phenotypic properties

**Figure 2:**
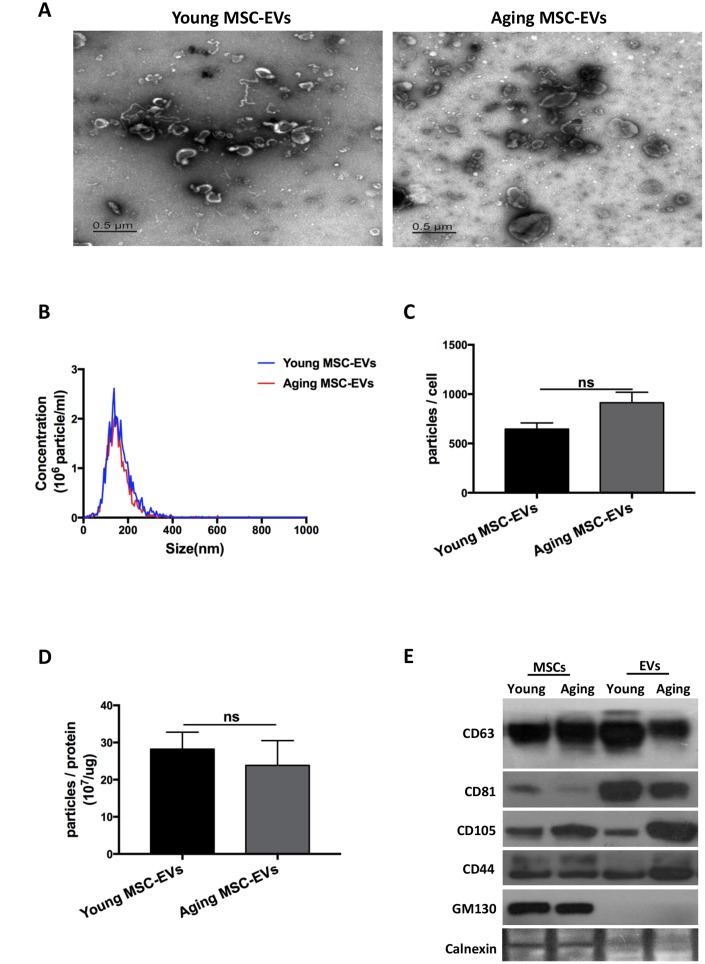
**EVs derived from young and aging human adipose-derived MSCs showed similar characteristics.** (**A**) Morphology of the isolated young and aging MSC-EVs as imaged by transmission electron microscopy (scale bar: 0.5 μm). (**B**–**D**) Size and particle concentrations of isolated young and aging MSC-EVs were examined via nanoparticle tracking analysis. Data are presented as mean ± SEM, n = 4–7. (**E**) Markers for MSC-EVs were assayed by Western blot analysis, n = 3.

Aging and young MSC-EVs were isolated from MSC culture medium via differential ultracentrifugation for characteristic analysis. Electron microscopy revealed that aging and young MSC-EVs had similar spherical sizes (Figure 2A). Data of nanoparticle tracking analysis showed that both aging and young MSC-EVs had similar size and distribution (Figure 2B). Young and aging MSC- EVs had similar particles/cell ratio (646/cell vs 913/cell, p > 0.05, Figure 2C) and particles/protein ratio (2.8 × 10^8^/μg vs 2.4 × 10^8^/μg, p > 0.05, Figure 2D). In addition, both young and aging MSCs-EVs expressed markers for EVs (CD63 and CD81) and MSCs (CD105 and CD44) in Western blots. Furthermore, both MSC-EVs were negative for GM130 (Golgi marker) and calnexin (endoplasmic reticulum marker) (Figure 2E). These results demonstrate that both aging and young MSC-EVs share many physical and phenotypic characteristics.

### Aging and young MSC-EVs exhibit differential effects in acute lung injury

**Figure 3:**
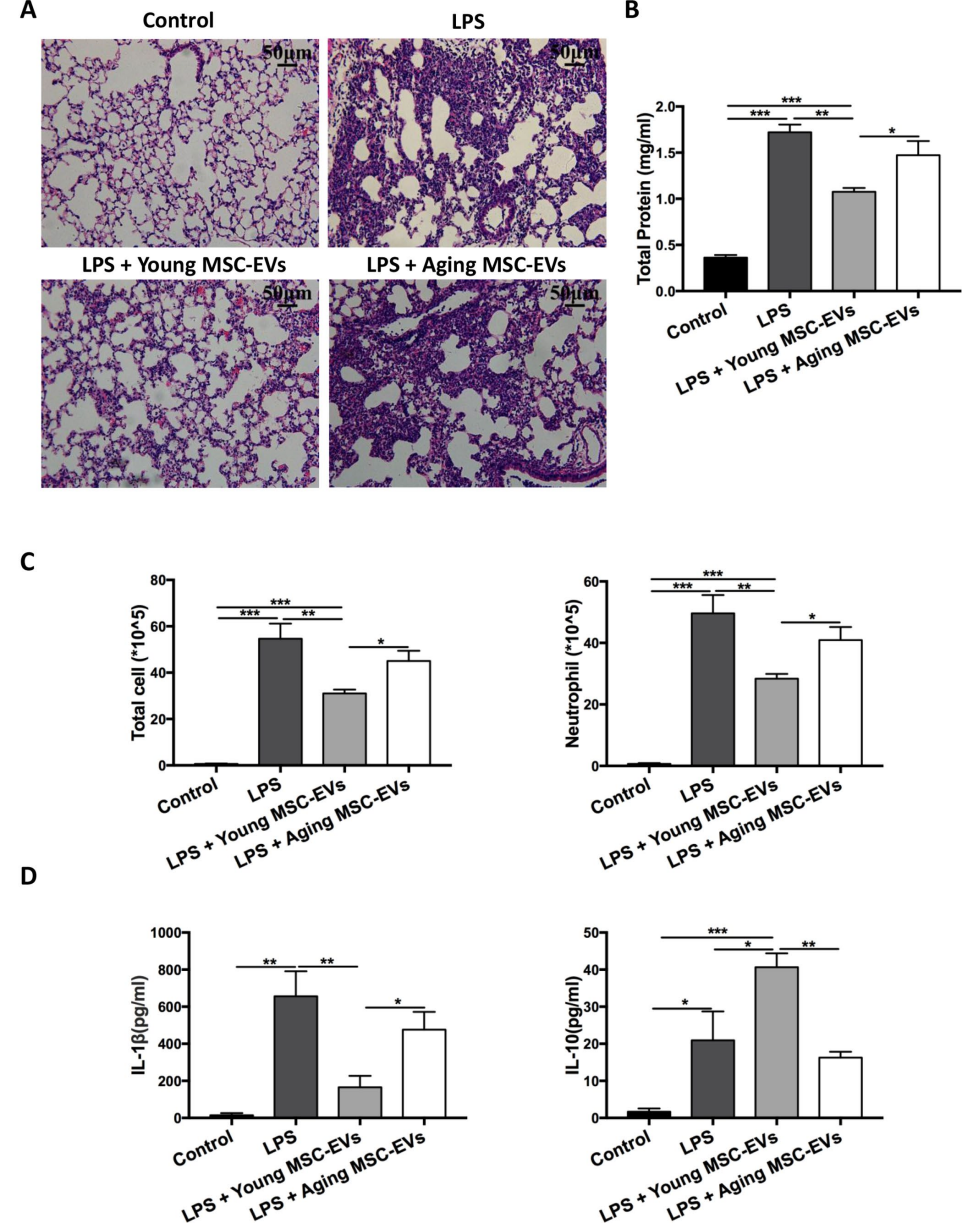
**Aging MSC-EVs failed to alleviate LPS-induced lung injury.** Mice were divided into 4 groups: control, LPS, LPS + young MSC-EVs (100 μg), LPS + aging MSCs-EVs (100 μg). MSC-EVs were administered intravenously 30 min after intratracheal LPS treatment. (**A**) Lung tissue was harvested at 48 h after LPS treatment, stained with H&E, and visualized at x200 magnification (scale bar: 50 μm). (**B** and **C**) Protein level, total cell count, and neutrophil count in the BAL were examined at 48 h after LPS treatment to evaluate inflammatory response. Data are presented as mean ± SEM, n = 8. *p < 0.05, **p < 0.01, *** *p* < 0.001. (**D**) Cytokine levels (IL-1β and IL-10) in the BAL at 48 h after LPS treatment were assayed via ELISA. Data are presented as mean ± SEM, n = 4-5. *p < 0.05, **p < 0.01, *** *p* < 0.001.

To determine whether aging and young MSC-EVs could mimic their parental cells in acute lung injury, aging and young MSC-EVs (100 μg in protein content) were administered intravenously to young mice with LPS-induced acute lung injury to compare their protective effects (control, LPS, LPS + young MSC-EVs, and LPS + aging MSC-EVs). In histology analysis, treatment of mice with young MSC-EVs reduced the inflammatory cell accumulation and alveolar septal thickness at 48 h post injury compared with the LPS group (Figure 3A). Compared with the LPS group, young MSC-EVs significantly reduced protein, total cells, and neutrophils (by 37.4%, 43.2%, 42.8%, respectively) in the BAL at 48 h (Figure 3B and 3C). Young MSC-EVs also reduced proinflammatory cytokine IL-1β and raised anti-inflammatory IL-10 in the BAL at 48 h (Figure 3D). Similar to their parental cells, aging MSC-EVs failed to display the beneficial effects in acute lung injury compared with young MSC-EVs.

### Young MSC-EVs rather than aging MSC-EVs favor M2 macrophages *in vivo*

**Figure 4:**
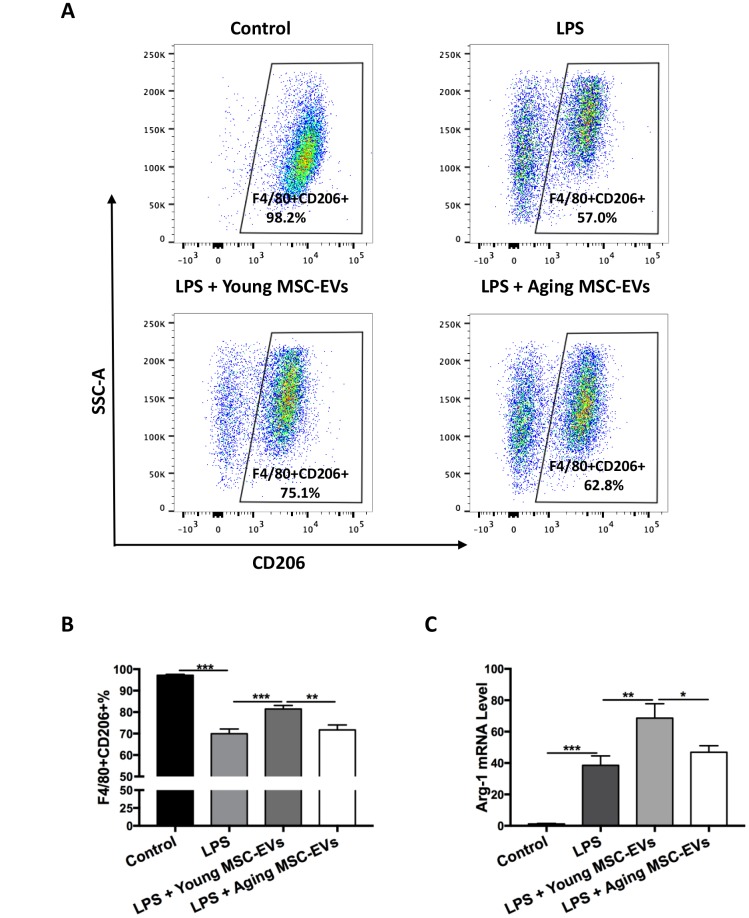
**Young MSC-EVs, but not aging MSC-EVs, promoted M2 phenotype of alveolar macrophages *in vivo*.** Mice were divided into 4 groups: control, LPS, LPS + young MSC-EVs (100 μg), LPS + aging MSCs-EVs (100 μg). (**A** and **B**) Total cells in the BAL were harvested at 24 h after LPS treatment. Cells were then stained with PE-conjugated anti-mouse F4/80 antibody and APC anti-mouse CD206 antibody for phenotypical analysis via flow cytometry. Data are presented as mean ± SEM, n = 8–12. **p < 0.01, *** *p* < 0.001. (**C**) Total cells in the BAL were harvested to isolate alveolar macrophages at 24 h after LPS treatment. Arg-1 mRNA levels of alveolar macrophages were analyzed via qRT-PCR. Data are presented as mean ± SEM, n = 6. *p < 0.05, **p < 0.01, *** *p* < 0.001.

In order to study the effects of young and aging MSC-EVs on macrophage polarization, BAL were harvested from different groups of mice as described in the previous paragraph (control, LPS, LPS + young MSC-EVs, and LPS + aging MSC-EVs) at 24 h after LPS treatment. BAL was first analyzed phenotypically via flow cytometry. Flow cytometry analysis demonstrated that young MSC-EVs increased the expression of F4/80+CD206+ cells compared with the LPS group, favoring the orientation toward M2 macrophages, whereas aging MSC-EVs had a significantly diminished effect in M2 phenotype compared with young MSC-EVs (Figure 4A and 4B). Then, BAL macrophages were separated and analyzed for level of arginase-1 (Arg-1), a marker for M2 macrophages. Young MSC-EVs raised the mRNA level of Arg-1 in contrast to both LPS treatment and aging MSC-EVs (Figure 4C).

### Young MSC-EVs reduce macrophage recruitment *in vivo*

**Figure 5:**
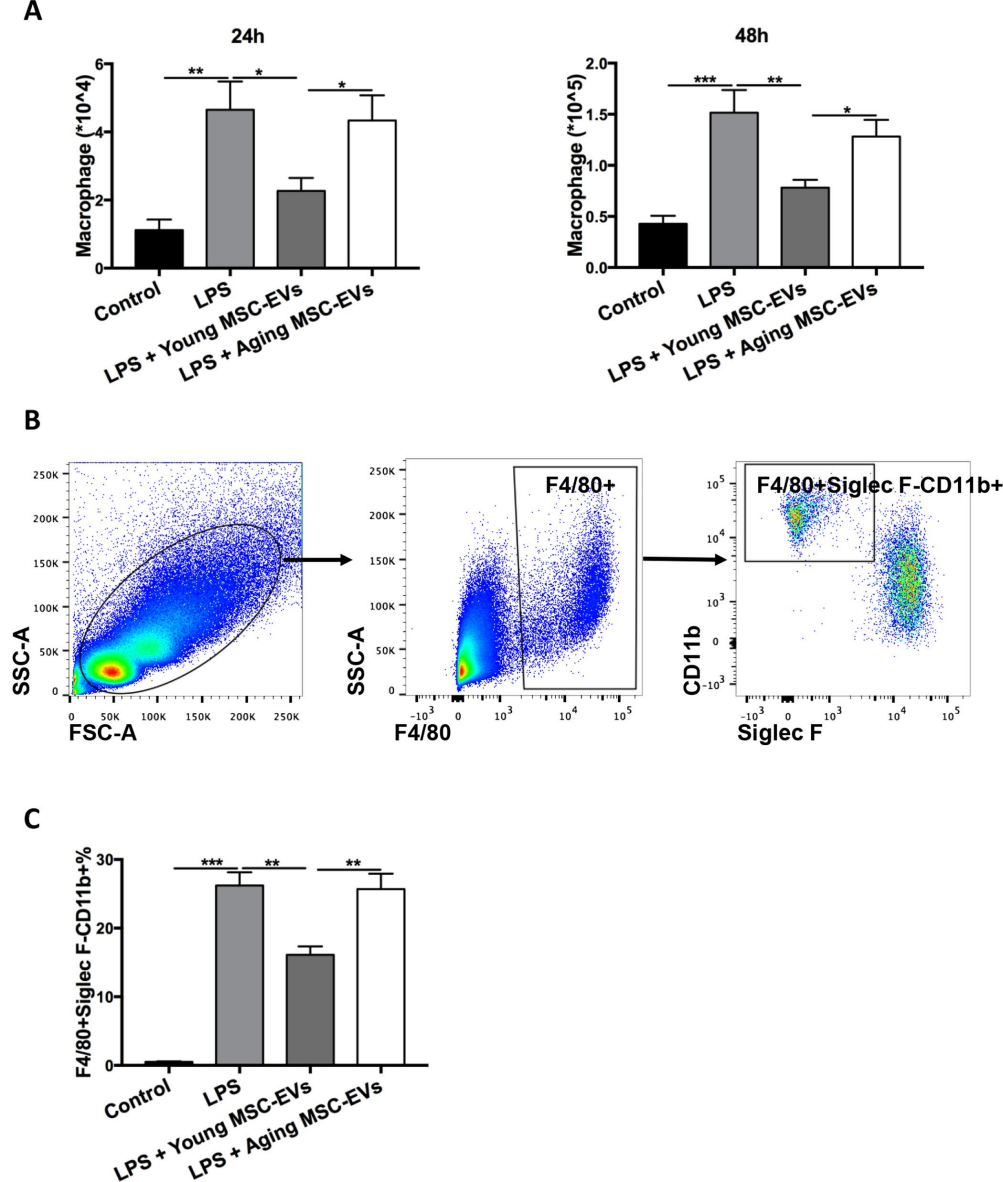
**Young MSC-EVs diminished macrophage recruitment *in vivo*.** Mice were divided into 4 groups: control, LPS, LPS + young MSC-EVs (100 μg), LPS + aging MSCs-EVs (100 μg). (**A**) Total cells in the BAL were harvested at 24 h and 48 h after LPS treatment. Total macrophages were quantitated by percentage of F4/80+ macrophages multiplying by total cells in BAL. (**B**) Dot plots represent gating strategy to stain macrophage subpopulations from BAL of LPS-treated mice. Macrophages were identified as F4/80+. Recruited macrophages were defined as F4/80+Siglec F-CD11b+. (**C**) Percentage of recruited macrophages in BAL at 24 h was analyzed via flow cytometry. Data are presented as mean ± SEM, n = 8–12. *p < 0.05, **p < 0.01, *** *p* < 0.001.

To study the effects of young and aging MSC-EVs on macrophage recruitment, BAL was harvested from the above experiment at 24 h and 48 h after LPS treatment and the number of F4/80+ macrophages were examined. Young MSC-EVs decreased the total macrophages in BAL compared with LPS group, while aging MSC-EVs did not produce such effect (Figure 5A). In order to further quantify recruited macrophages in BAL, cells were analyzed via flow cytometry. F4/80+Siglec F-CD11b+ cells were designated as recruited macrophages (Figure 5B) as described by Smith et al. [22]. At 24 h post injury, young MSC-EVs significantly reduced the percentage of recruited macrophages compared with LPS treatment, while aging MSC-EVs had no such effect (Figure 5C).

### Aging MSC-EVs fail to alter macrophage phenotypes *in vitro*

**Figure 6:**
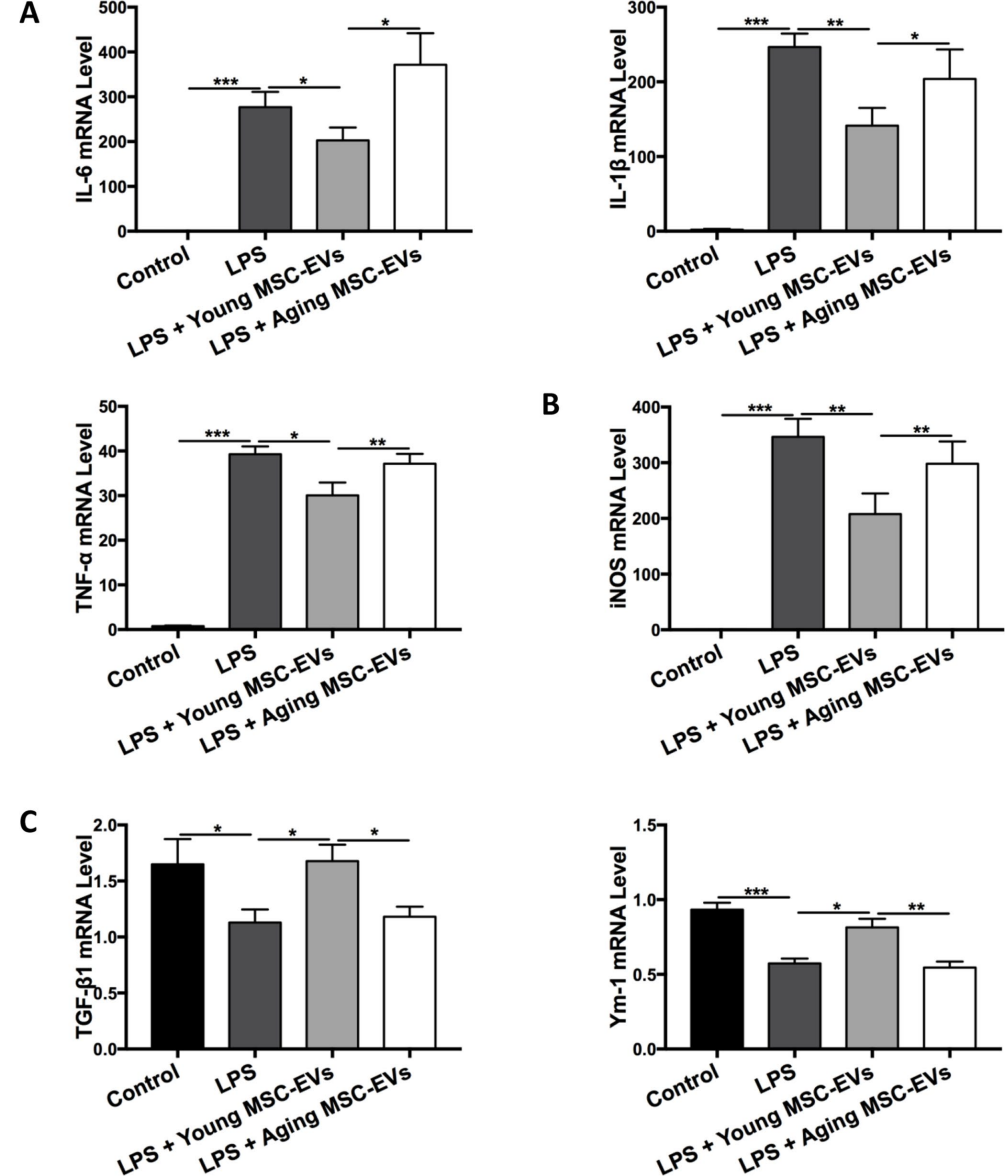
**Aging MSC-EVs were incapable of modulating macrophage polarization *in vitro*.** (**A**–**C**) BMDMs (3 × 10^5^ cells) were cultured with young or aging MSC-EVs (100 μg/ml) in a 24-well plate in the presence or absence of LPS (100 ng/ml). Cells were collected after 24 h and mRNA levels of pro-inflammatory cytokines IL-6, IL-1β, and TNF-α (**A**), M1 marker iNOS (**B**), and M2 markers TGF-β1 and Ym-1 (**C**) were analyzed via qRT-PCR. Data are presented as mean ± SEM, n = 6–8. *p < 0.05, **p < 0.01, *** *p* < 0.001.

To corroborate the differential effects on modulating macrophage phenotypes between young and aging MSC-EVs, bone marrow-derived macrophages (BMDMs) were cultured with young or aging MSC-EVs and stimulated with LPS for 24 h. Young MSC-EVs (100 μg/ml) suppressed the activation of proinflammatory genes IL-6, IL-1β, TNF-α induced by LPS as determined by qRT-PCR, while aging MSC-EVs (100 μg/ml) had no effect (Figure 6A). Young MSC-EVs (100 μg/ml), but not aging MSC-EVs (100 μg/ml), decreased the expression of M1 macrophage marker iNOS induced by LPS (Figure 6B) while simultaneously enhanced M2 markers TGF-β1 and Ym-1 (Figure 6C). Culture of BMDMs with higher dose of aging MSC-EVs (400 μg/ml) was unable to mimic the effects of young MSC-EVs (100 μg/ml) (data not shown), suggesting aging MSC-EVs may lack the potency in altering macrophage phenotypes *in vitro*.

### Aging MSC-EVs have lower efficacy in internalization by macrophages

**Figure 7:**
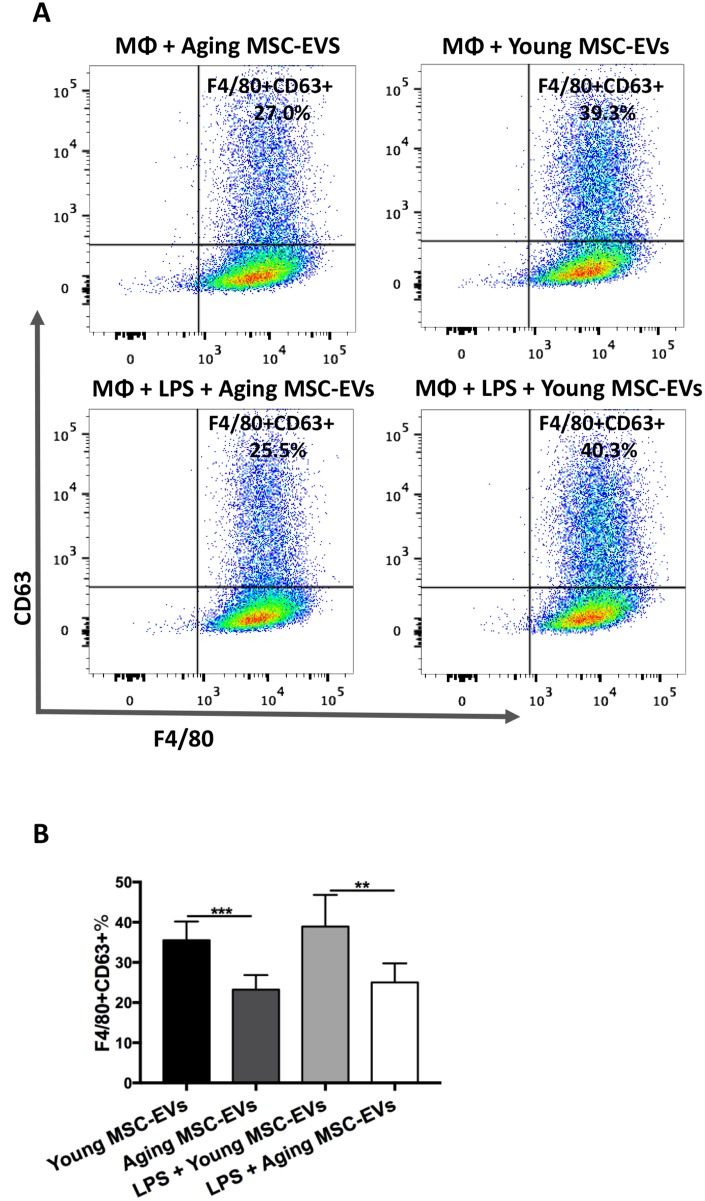
**Aging MSC-EVs were less readily internalized by macrophages compared with young MSC-EVs.** (**A**–**B**) BMDMs (8 × 10^5^ cells) were incubated with CD63-labelled aging or young MSC-EVs (10 μg) in a 12-well plate for 6 h in the presence or absence of LPS and stained with PE-Cy7-conjugated anti-mouse F4/80 antibody. Data were analyzed via flow cytometry. Data are presented as mean ± SEM, n = 6. **p < 0.01, *** *p* < 0.001. MΦ = Macrophages.

The uptake of protein and nucleic acids from EVs by target cells has been proposed as a novel mechanism of intercellular communication. Therefore, the uptake by macrophages was compared between aging and young MSC-EVs. EVs were labeled with CD63 and cultured with BMDMs for 6 h to examine internalization via flow cytometry. The percentage of macrophages with internalized EVs (F4/80+CD63+) was significantly lower in aging MSC-EVs group compared with young counterpart, indicating lower efficacy in internalization (Figure 7A and 7B). Furthermore, LPS stimulation did not alter the internalization by macrophages for both aging and young MSC-EVs (Figure 7A and 7B).

### Young and aging MSC-EVs have differential expression in miRNAs

**Figure 8:**
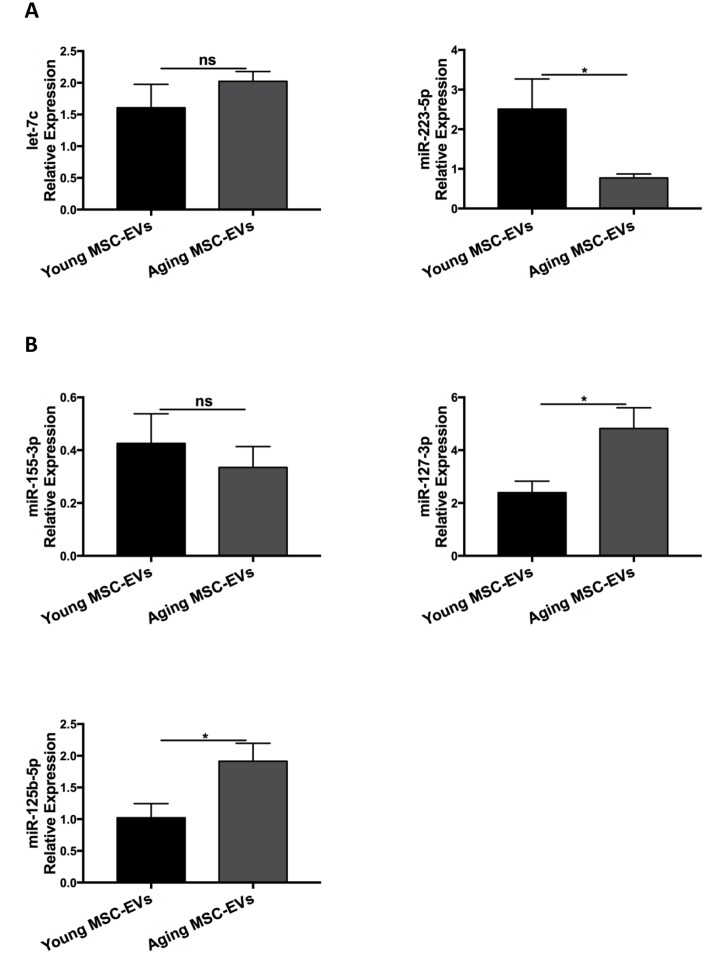
**Young and aging MSC-EVs expressed differential levels of miRNAs.** (**A** and **B**) Young and aging MSC-EVs were isolated from supernatants of MSCs via ultracentrifugation and levels of miRNAs associated with macrophage polarization (let-7c, miR-223-5p, miR-155-3p, miR-127-3p, and miR-125b-5p) in MSC-EVs were analyzed via qRT-PCR. All miRNAs were standardized to U6 snRNA using standard ΔΔCt method. Data are presented as mean ± SEM, n = 4–8. *p < 0.05.

MSC-EVs play a role in intercellular communication via transfer of their contents, including mRNA, miRNA, and proteins. Several miRNAs have been reported to mediate M1 or M2 macrophage polarization. To explore the mechanism underlying the differential effects between young and aging MSC-EVs in macrophage polarization, MSC-EVs were examined for miRNAs associated with M2 polarization (let-7c, miR-223-5p) (Figure 8A) and M1 polarization (miR-155-3p, miR-127-3p, miR-125b-5p) (Figure 8B) via qRT-PCR. There was no significant difference in let-7c and miR-155-3p levels between young and aging MSC-EVs. Young MSC-EVs showed higher expression of miR-223-5p and lower levels of miR-127-3p and miR-125b-5p compared with aging MSC-EVs.

### Inhibition of miR-127-3p and miR-125b-5p mitigates M1 macrophage polarization

To examine whether reduced expression of miR-127-3p and miR-125b-5p alters macrophage polarization, BMDMs were transfected with inhibitors for miR-127-3p or miR-125b-5p. Six hours after the transfection, BMDMs were treated with LPS (100 ng/mL) for 24 hours.

**Figure 9:**
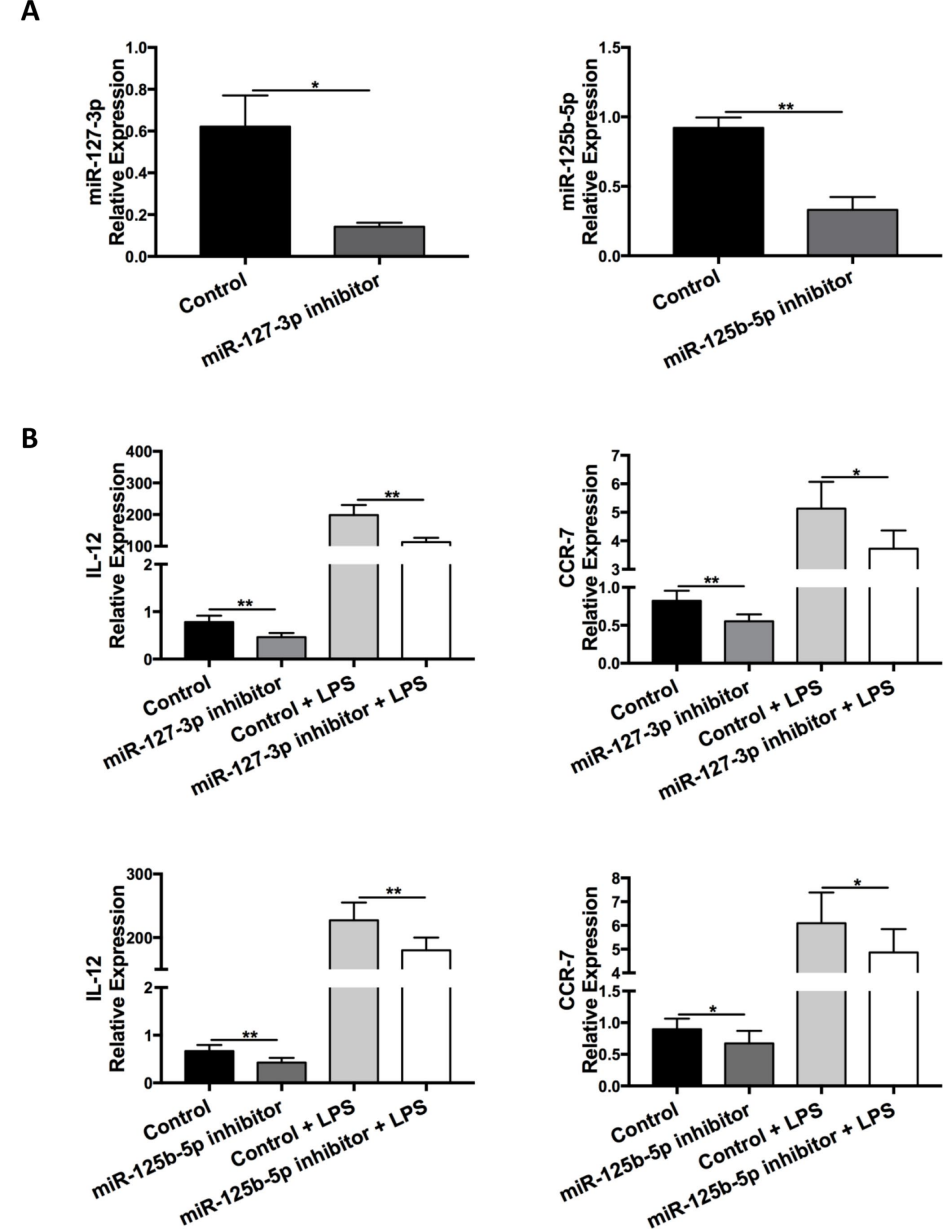
**Inhibition of miR-127-3p and miR-125b-5p decreased M1 macrophage polarization.** BMDMs were transfected with miR-127-3p inhibitor/control RNA or miR-125b-5p inhibitor/control RNA and stimulated with or without LPS (100 ng/ml) 6 h later. (**A**) Confirmation of successful transfection of miR-127-3p inhibitor and miR-125b-5p inhibitor was determined via qRT-PCR. Data are presented as mean ± SEM, n = 4-5. *p < 0.05, **p < 0.01. (**B**) Levels of the M1 markers IL-12 and CCR-7 in BMDMs at 24 h after LPS treatment were analyzed via qRT-PCR. Data are presented as mean ± SEM, n = 6–10. *p < 0.05, **p < 0.01.

miR-127-3p and miR-125b-5p levels after transfection were determined via qRT-PCR (Figure 9A). Transfection of miR-127-3p and miR-125b-5p inhibitors reduced the expression of M1 markers IL-12 and CCR-7 with or without LPS treatment (Figure 9B). On the other hand, BMDMs were transfected with miR-223-5p mimic and examined for macrophage polarization. However, miR-223-5p mimic did not affect the expression of M1 or M2 markers (data not shown).

## DISCUSSION

The present study demonstrates that aging MSC-EVs have impaired therapeutic effects in a murine model of LPS-induced acute lung injury compared with young MSC-EVs. Aging MSC-EVs are deficient in promoting M2 macrophage polarization and decreasing macrophage recruitment. Mechanistically, aging MSC-EVs are less readily internalized by macrophages compared with young counterparts. In addition, young and aging MSC-EVs have differential expression in miRNAs associated with macrophage polarization.

Accumulating evidences support that MSC-EVs, typically from young donors, could be a novel option for treating acute lung injury from diverse etiologies. MSC-EVs were first reported to reduce LPS-induced acute lung injury via KGF mRNA [16]. Then, MSC-EVs were shown to alleviate lung injury in a mouse model of E. coli-induced pneumonia via uptake of EVs through the CD44 receptor [17]. Potter et al. found that MSC-EVs alleviated lung injury induced by hemorrhagic shock via reducing pulmonary vascular permeability [23]. Porzionato et al. discovered that intratracheal administration of MSC-EVs ameliorated lung injury in a hyperoxia-induced model of bronchopulmonary dysplasia [24]. Stone et al. documented that MSC-EVs mitigated lung ischemia-reperfusion injury and improved reconditioning of donor lungs via reducing immune cell activation [25]. Khatri et al. reported that MSC-EVs had anti-influenza and anti-inflammatory properties and alleviated influenza virus-induced lung injury in a pig model [26]. In addition, Ahn et al. revealed that vascular endothelial growth factor mediated the therapeutic effects of MSC-EVs against neonatal hyperoxic lung injury [27]. Furthermore, Morrison et al. showed that adoptive transfer of macrophages pretreated with MSC-EVs alleviated LPS-induced acute lung injury [11]. However, it is unknown whether aging MSC-EVs possess similar capacity as the young counterparts. The present study revealed that aging MSC-EVs lacked the therapeutic effects in a mouse model of LPS-induced acute lung.

Several studies have demonstrated that MSC-EVs exert their anti-inflammatory effects via inducing M2 polarization of macrophages [28, 29]. There is a list of miRNAs that are reported to mediate macrophage polarization. For example, miR-9, miR-155, miR-127, and miR-125b skew macrophages towards M1 phenotype, while let-7b, let-7c, miR-223, miR-34a, and miR-146a induce M2 polarization via various targets proteins [30]. Ti et al. reported that let-7b was shuttled from MSC-EVs to induce M2 polarization of macrophages and alleviate chronic inflammation [31]. Song et al. documented that miR-146a was transferred from MSC-EVs to macrophages, in polarization, and enhanced the therapeutic efficacy of MSCs against sepsis [32]. Our results showed that aging MSC-EVs had higher levels of miR-127-3p and miR-125b-5p (M1) compared with young MSC-EVs. This finding might explain the observed difference in M2 macrophage polarization between aging and young MSC-EVs.

Many studies have also revealed that the composition of EVs including DNA, mRNA, microRNA, and proteins changes with age, which might contribute to aging and immunomodulatory process. EVs from plasma and cerebrospinal fluid had differential changes in IL-1β and CD63 levels in aged rats [33]. Higher levels of C24:1 ceramide were found in serum EVs from older women compared with young women. Furthermore, serum EVs loaded with C24:1 ceramide were able to induce senescence in MSCs [34]. Mitsuhashi et al. reported that higher levels of IL-6, TNF-α, and IL-12 mRNAs were detected in EVs from LPS or Amyloid-β1-42-stimulated macrophages from old subjects in comparison to young counterparts [35]. Wang et al. documented that MSC-EVs from older rats were weaker in inhibiting epithelial-mesenchymal transition than younger rats. In addition, the expression for miR-133b-3p and miR-294 were downregulated in older rats [36]. Lei et al. found that late passage of MSC-EVs and MSCs had elevated expression of miR-146a-5p and down-regulation of its target genes [37]. Terlecki-Zaniewicz et al. identified senescence-specific alterations in miRNAs of EVs from human dermal fibroblasts, with an elevation of miR-23a-5p and miR-137 and a reduction of miR-625-3p, miR-766-3p, miR-199b-5p, miR-381-3p, miR-17-3p [38]. Fafián-Labora et al. compared expression of miRNAs in MSC-EVs from different age groups. They discovered that levels of miR-146a, miR-155 and miR-132 decreased significantly with increasing donor age. The adult group showed the highest expression of miR-335, while the pre-pubertal group presented the highest level of miR-21 with respect to the other groups [39]. Therefore, it is reasonable to propose that the observed alteration of miRNA levels in the present study might serve as a marker for aging.

The effects of EVs rely on internalization and subsequent release of their contents in target cells. EVs can gain entry into target cells via different mechanisms, including clathrin-dependent endocytosis and clathrin-independent mechanisms such as caveolar endocytosis, macropinocytosis, and phagocytosis [40]. Furthermore, EVs may be internalized via a combination of mechanisms due to their heterogeneity in size [41]. Internalization and functional activity of cancer EVs were shown to rely on heparan sulfate proteoglycans of target cells as receptors [42]. Gonda et al. reported that survivin in cancer EVs facilitated vesicle internalization. EV internalization was reduced when survivin was inhibited [43]. Eitan et al. found that plasma EVs from aging individuals were more easily internalized by B cells [44]. In the present study, we found that aging MSC-EVs had lower efficacy in internalization by macrophages. Further studies are warranted to define the mechanisms through which MSC-EVs ameliorate acute lung injury.

In conclusion, although both aging and young MSC-EVs have similar physical and phenotypical characteristics, only young MSC-EVs are able to alleviate LPS-induced acute lung injury and alter macrophage phenotypes. The observed differences may be explained by differential miRNA expression and internalization of the EVs. Our data suggest that aging MSC-EVs are not suitable for the treatment of acute lung injury.

## MATERIALS AND METHODS

### MSCs culture and isolation of EVs

The study was conducted in adherence with the Declaration of Helsinki and was pre-approved by the Research Ethics Committee at Shaoxing Second Hospital. Young and aging human adipose-derived mesenchymal stem cells were isolated from lipo-aspirates of healthy donors of 25 (n=1) and 72 (n=1) years old, respectively, with informed consents. Adipose tissue was extensively washed with cold phosphate-buffered saline (PBS) until a clear solution was obtained. The adipose tissue sample was digested with 0.1% collagenase IA (Sigma, St. Louis, MO) in PBS at 37°C for 60 min and centrifuged at 300 g for 5 min at 4 °C. The cell pellets were resuspended with expansion medium consisted of Dulbecco’s modified Eagle medium (DMEM, Biological Industries, Cromwell, CT) low glucose supplemented with 10% EV-depleted fetal bovine serum (Biological Industries, Cromwell, CT) obtained from ultracentrifugation (118,000 g for 16 h at 4 °C) and penicillin/streptomycin antibiotics (Thermo Fisher Scientific). The cells were cultured in a humidified incubator at 37°C with 5% CO_2_ until approximately 80% confluence. Cells were then treated with 0.5 % trypsin-EDTA and re-plated at 1 × 10^4^ cells/cm^2^ for more passages. The aged donor had reduced yield of MSCs compared with the young donor. Aged MSCs displayed decreased proliferation with elevated doubling time. These findings are in consistent with previously published reports [45]. The resulting MSCs in passages 4–5 were used for isolation of EVs. To harvest EVs, the culture medium was collected and centrifuged sequentially for 15 min at 1500 g and 30 min at 16,500 g, followed by centrifugation for 2 h at 118,000 g at 4°C in a swinging bucket rotor (Optima XPN- 80, SW 32 Ti rotor, Beckman Coulter). The resulting pellet was resuspended in 100–200 μl of PBS and stored at -80°C freezer for further experiments. BCA Protein Assay Kit (ThermoFisher Scientific) was used to quantify the total protein concentration of isolated EVs for in *in vivo* and *in vitro* assays.

### Transmission electron microscopy

Physical characterization of EVs was performed via transmission electron microscopy. Fresh EVs samples were resuspended in 100 μl of PBS. Samples were absorbed onto 200 mesh formvar copper grids. Then, the grids were negatively stained with 2% aqueous uranyl acetate for 30 sec and dried in air. The grids were observed on a transmission electron microscope (FEI Tecnai Spirit G2) to view the EVs.

### Nanoparticle tracking analysis

The size distribution and particle concentration of EVs were measured using nanoparticle tracking analysis via ZetaView PMX 110 (Particle Metrix, Meerbush, Germany). Samples were diluted into appropriate concentrations using PBS and measured according to the operating instructions.

### Western blot

For phenotypic characterization, MSCs and MSC-EVs were lysed via lysis buffer (10 mM Tris-HCl, pH 7.4, 150 mM NaCl, 0.5% Nonidet P-40, 1 mM EDTA, 1 mM Na_3_VO_4_, and 1 mM PMSF). Equal amount of total proteins from samples (20 μg or 40 μg) were separated by 12% sodium dodecyl sulfate-polyacrylamide gel electrophoresis (SDS-PAGE), transferred to polyvinylidene fluoride membranes (Millipore, Billerica, MA, USA), and detected via Western blot analysis. The primary antibodies used in the study included the following: CD63 (0.5 μg/ml; ab193349, Abcam, Cambridge, MA), CD81 (1:1000; sc-166029, Santa Cruz Biotechnology, Santa Cruz, CA), CD105 (1:1000; ab169545, Abcam), CD44 (1:5000; ab157107, Abcam), GM130 (1:5000; ab52649, Abcam), and Calnexin (1:5000; ab133615, Abcam). After washing, membranes were incubated with secondary antibodies conjugated to horseradish peroxidase. Goat anti-Rabbit lgG (H+L) HRP Conjugated (1:5000, GAR0072, Multi Sciences, Hangzhou, China) and goat anti-Mouse lgG (H+L) HRP Conjugated (1:5000, GAM0072, Multi Sciences, Hangzhou, China) were used as Secondary antibodies. The signals were detected via enzyme-linked chemiluminescence using the EZ-ECL kit (Biological Industries, Kibbutz Beit-Haemek, Israel).

### LPS-induced acute lung injury and harvesting alveolar macrophages from BAL

C57BL/6 mice (6–8 weeks old; Shanghai Laboratory Animal Center, Shanghai, China) were used for the *in vivo* study to mimic adult ARDS. All mice were housed in the Zhejiang University Laboratory Animal Center. Animal experiment protocols were approved by the review committee from Zhejiang University School of Medicine and were in compliance with institutional guidelines. A total of two hundred-thirty mice were used for the *in vivo* studies, while forty mice were used for the *in vitro* experiments (for BMDMs). Mice were anesthetized with 4% trichloroacetaldehyde intraperitoneally and instilled intratracheally with LPS from Escherichia coli O111:B4 (Sigma, St. Louis, MO, 4 mg/kg) or PBS as control. Young or aging MSCs (1 × 10^6^ cells/200ul) and MSC-EVs (100 μg/200ul) were administered 30 min after LPS insult via tail vein. Animals were sacrificed at 24 h or 48 h after LPS or PBS treatment. Lungs and BAL samples were collected from animals for further analysis. To separate alveolar macrophages, BAL was centrifuged at 400 g for 5 min at 4 °C. The cell pellet was resuspended with RPMI-1640 and plated 2 ml into each 6-well culture dish. The cells were then allowed to adhere to the bottom of the wells by incubating in a 37 °C humidified incubator with 5% CO2 for 30 minutes. Non-adherent cells and debris were removed by changing the culture medium. The adherent cells were harvested for further experiment. Approximately 90% of the adherent cells were positive for macrophage markers.

### Primary culture of bone marrow-derived macrophages (BMDMs)

For BMDMs, bone marrow cells were obtained from C57BL/6 mice (6–8 weeks old) by flushing the femurs and tibias with cold PBS. After red blood cells were removed by treating with 1x lysis buffer (10 mM KHCO3, 155 mM NH4Cl, 0.1 mM EDTA) for 3 min, cells were washed with PBS and resuspended in culture medium containing DMEM supplemented with 20 ng/ml GM-CSF (PeproTech, Rock Hill, NJ), 10% fetal bovine serum, and 1% penicillin plus streptomycin at 37 °C with 5% CO2 overnight. Non-adherent bone marrow cells were collected and seeded on 6-well culture plates (2 × 10^6^ cells/well). Fresh culture medium was changed every 3 days. Adherent BMDMs were dissociated with lidocaine/EDTA (0.4% lidocaine, 5 μM EDTA) after 7 days, counted, and reseeded onto cell culture plates for further experiments.

### EV-labelling and uptake assay

First, both young and aging MSC-EVs (10 μg) were blocked with 5% bovine serum albumin for 1 h, washed with PBS, and ultra-centrifuged at 118,000g for 2 h at 4 °C. After washing, EVs were incubated with or without mouse anti-CD63 antibody (Abcam, Cambridge, MA) at 1 μg/ml overnight and ultra-centrifuged at 118,000g for 2 h at 4°C. Then, pellets were incubated with cy3-conjugated donkey anti-mouse secondary antibody (1:100, MilliporeSigma, Burlington, MA) for 2 h, washed with PBS, and ultra-centrifuged at 118,000 g at 4°C for 2 h. For uptake studies, CD63-labelled EVs were cultured with BMDMs (8 × 10^5^ cells/well, 12-well plate) in serum free media. After 6 h, BMDMs were harvested and stained with PE-Cy7-conjugated anti-mouse F4/80 (ThermoFisher Scientific). Cells were analyzed via flow cytometry (BD LSRFortessa™).

### Culture of BMDMs with EVs in vitro

Mouse BMDMs were dissociated and reseeded on a 24-well plate at 3 × 10^5^ cells per well. Cells were cultured alone or with young or aging MSC-EVs and simultaneously stimulated with LPS from Escherichia coli 055:B5 (Sigma, St. Louis, MO, 100 ng/mL). After 24 h, cells were collected for mRNA expression detection.

### Transfection of inhibitors for miRNAs into BMDMs

Inhibitors/controls for miR-125b-5p and miR-127-3p were purchased from GenePharma (Shanghai, China). BMDMs were transfected with inhibitors for miR-125b-5p and miR-127-3p (20 μM) using lipofectamine RNAiMAX reagent (invitrogen) following the manufacturer’s procedure. Six hours after the transfection, BMDMs were stimulated with LPS (100 ng/mL) for 24 hours. BMDMs were harvested for qRT-PCR analysis to exam markers for M1/M2 macrophages. The efficiency of the transfection was confirmed via qRT-PCR.

### Histopathology

To harvest the lungs, the tracheas were cannulated and the lungs fixed by inflation with 4% paraformaldehyde. After 24 h fixation, lungs were embedded in paraffin and sectioned at 5 μm in thickness. Hematoxylin and eosin (H&E) staining was performed to determine morphology and inflammatory infiltration. Olympus BX53 microscope (Shinjuku, Tokyo, Japan) was used for imaging.

### Inflammatory cell counts, protein and cytokine in BAL

To obtain BAL cells from mice, lungs were lavaged three times with 0.4 ml of cold PBS. Total cell counts in the BAL were counted via a hemocytometer. BAL was further labeled with FITC-conjugated anti-mouse Ly-6G (Gr-1) antibody (Thermo Fisher Scientific) to determine the percentage of neutrophil via flow cytometry. Neutrophil counts were calculated by multiplying the percentage of neutrophil by the total number of total cells in the BAL. BAL was then centrifuged at 800g for 5 min to collect supernatant for analysis of total protein and cytokine levels. Protein concentration in BAL was determined using a BCA Protein Assay Kit. Cytokine levels were analyzed via ELISA for IL-1β (ThermoFisher Scientific) and IL-10 (R&D Systems, Minneapolis, MN) according to the manufacturer’s protocol. OD values of the plates were detected by using SpectraMax 190 Microplate Reader (Molecular Devices, San Jose, CA).

### Real-time quantitative reverse-transcriptase polymerase chain reaction (qRT-PCR)

Total RNA was isolated from cells or MSC-EVs using TRIzol reagent (ThermoFisher Scientific). For mRNA analysis, reverse transcription reaction was performed via PrimeScript^TM^ RT Reagent Kit (Takara Bio, Kusatsu, Japan) according to the manufacturer’s instructions. qRT-PCR was performed using SYBR Green^TM^ Premix Ex Taq^TM^ (Takara Bio) on LightCycler 480 II (Roche). miRNA was determined using Mix-X^TM^ miRNA First Strand Synthesis Kit (Takara Bio) followed by LightCycler 480 II via Mir-X miRNA qRT-PCR SYBR Kit (Takara Bio). All target genes were standardized to β-Actin for mRNA or U6 snRNA for miRNA using standard ΔΔCt method. The primer sequences used were listed as following: arginase-1 (Arg-1) forward 5′CTCCAAGCCAAAGTCCTTAGAG3′, reverse 5′GG AGCTGTCATTAGGGACATCA3′; IL-6 forward 5′CT GCAAGAGACTTCCATCCAG3′, reverse 5′AGTGGT ATAGACAGGTCTGTTGG3′; IL-1β forward 5′GAAA TGCCACCTTTTGACAGTG3′, reverse 5′TGGATGCT CTCATCAGGACAG3′; TNF-α forward 5′CAGGCGG TGCCTATGTCTC3′, reverse 5′CGATCACCCCGAAG TTCAGTAG3′; iNOS forward 5′CAGGCTGGAAGCT GTAACAAAG3′, reverse 5′GAAGTCATGTTTGCCG TCACTC3′; TGF*-*β1 forward 5′GAGAGCCCTGGATA CCAACT3′, reverse 5′CAACCCAGGTCCTTCCTAAA 3′; YM-1 forward 5′CAGGTCTGGCAATTCTTCTGA A3′, reverse 5′GTCTTGCTCATGTGTGTAAGTGA3′; IL-12 p40 forward 5′ GTCCTCAGAAGCTAACCATC TCC3′, reverse 5′ CCAGAGCCTATGACTCCATGTC 3′; CCR-7 forward 5′ CAGGTGTGCTTCTGCCAAG AT3′, reverse 5′ GGTAGGTATCCGTCATGGTCT3′; β-actin forward 5′CGTTGACATCCGTAAAGACC3′, reverse 5′AACAGTCCGCCTAGAAGCAC3′; let-7c- 5p 5′TGAGGTAGTAGGTTGTATGGTTA3′; miR-223-5p 5′CGTGTATTTGACAAGCTGAGTTAAA3′; miR-155-3p 5′GCTCCTACATATTAGCATTAACAAAAA3′; miR-127-3p 5′GATCCGTCTGAGCTTGGCTAAA3′; miR-125b-5p 5′CCCTGAGACCCTAACTTGTGAAA3′; U6 5′TCGTGAAGCGTTCCATATTTTTAA3′.

### Flow cytometry for alveolar macrophages from in vivo studies

For determination of M2 macrophages and macrophage recruitment in BAL, cells were labelled with surface markers of PE anti-mouse F4/80 (BioLegend, San Diego, CA), BV421 anti-mouse Siglec F (BD, New York), and BV711 anti-mouse CD11b (BD), and APC anti-mouse CD206 (Invitrogen). Cells were then washed and resuspended with PBS for flow cytometry analysis. All data were collected on a flow cytometer (BD LSRFortessa™) and analyzed using FlowJo vX software.

### Statistical methods

Data are expressed as mean ± standard error of the mean (SEM). Statistical analysis was carried out using the GraphPad Prism software. Comparisons were analyzed by one-way ANOVA with a Bonferroni post hoc test or Student’s t-test. Values were considered significant if p < 0.05.
